# No Evidence for Association between Tyrosine Hydroxylase Gene Val81Met Polymorphism and Susceptibility to Tardive Dyskinesia in Schizophrenia

**DOI:** 10.4306/pi.2009.6.2.108

**Published:** 2009-06-30

**Authors:** Heon-Jeong Lee, Seung-Gul Kang, Jung-Eun Choi, Young-Min Park, Se-Won Lim, Min Kyu Rhee, Seung-Hyun Kim, Leen Kim

**Affiliations:** 1 Department of Psychiatry, Korea University College of Medicine, Seoul, Korea.; 2 Division of Brain Korea 21 Biomedical Science, Korea University College of Medicine, Seoul, Korea.; 3 Department of Psychiatry, Inje University College of Medicine, Goyang, Korea.; 4 Department of Psychiatry, Sungkyunkwan University College of Medicine, Seoul, Korea.; 5 Department of Psychology, Gyeongsang National University, College of Medicine, Jinju, Korea.

**Keywords:** Tardive dyskinesia, Tyrosine hydoxylase, Polymorphism

## Abstract

**Objective:**

Tyrosine hydroxylase (TH) is the rate-limiting enzyme in dopamine biosynthesis. Because the TH Val81Met polymorphism is located in the amino-terminal regulatory domain of the tetrameric enzyme, it is a candidate marker for susceptibility to dopamine-related traits. We investigated the hypothesis that TH Val81Met polymorphism can influence susceptibility to tardive dyskinesia (TD) in schizophrenia.

**Methods:**

TH Val81Met polymorphism was analyzed by PCR-based methods in 83 schizophrenic patients with TD and 126 schizophrenic patients without TD, matched for antipsychotic drug exposure and other relevant variables.

**Results:**

There was no significant association of the genotype and allele frequencies determined by the TH Val81Met polymorphism between TD and non-TD patients. In addition, there was no significant difference in terms of total Abnormal Involuntary Movement Scale scores among the three genotype groups.

**Conclusion:**

Within the limitations imposed by the size of the clinical sample, these findings suggest that the Val81Met polymorphism of the TH gene does not contribute significantly to the risk for TD.

## Introduction

Tardive dyaskinesia (TD) is one of the most serious adverse effects of neuroleptics. The typical symptoms of TD are involuntary movements of the orofacial musculature, trunk and extremities. Given that only a small proportion of those treated with neuroleptics develops TD, individual vulnerabilities such as genetic predisposition are important. Genetic vulnerability to the development of TD has been suggested based on the results of studies in both animals^1^ and families.^2^ Several biological mechanisms of TD have been hypothesized, including dopamine receptor supersensitivity,^3^ gamma-aminobutyric acid insufficiency,^4^ and oxidative stress.^5^ However, the pathophysiology of TD is not well understood.

Tyrosine hydroxylase (TH) is the rate-limiting enzyme in dopamine biosynthesis.^6^,^7^ The Val81Met polymorphism of the TH gene is the most frequent known coding sequence variant located in the regulatory domain of the tetrameric enzyme.^8^ Under physiological conditions, the activity of TH is regulated by phosphorylation.^9^ Phosphorylated TH has enhanced efficacy in dopamine synthesis. Antipsychotic drugs have been reported to increase TH activity,^10^ likely through increases of phosphorylated TH via binding to dopamine D2 receptors.^11^

Recently, alpha methyl paratyrosine (AMPT), an inhibitor of TH, has been reported to be clinically efficacious in the treatment of antipsychotic-induced TD.^12^ Consequently, it has been suggested that the efficacy of AMPT in TD may be related to a downregulation of TH activity that may be increased by neuroleptic-induced effects on TH phosphorylation.^12^

Thus, functional polymorphism of TH can be regarded as one of the candidate markers for susceptibility to neuroleptic-induced TD. We investigated a possible association between Val81Met polymorphism in exon 3 of the TH gene and TD in a genetically homogenous Korean population.

## Methods

### Subjects and tardive dyskinesia assessment

All subjects were examined by trained psychiatrists using the Korean version of the Structured Clinical Interview for DSM-IV, leading to a diagnosis based on DSM-IV criteria.^13^ Written informed consents were obtained and the study protocol was approved by the Ethics Committee of the Korea University Medical Center. None of the patients had a significant comorbid neurological illness, mental retardation or substance abuse, and all of them were inpatients at the three collaborating hospitals of Korea University hospital. Some findings from these subjects have been reported previously.^14^-^19^ Identification of potential recruits to the study was made via assessment with the Abnormal Involuntary Movement Scale (AIMS); 83 schizophrenic patients with TD were thus characterized.^20^ We included TD patients who had received atypical antipsychotics treatment (23 patients) and less than 10 years of treatment (18 patients) in our sample; however, all of the non-TD patients had received typical antipsychotic treatment for at least 10 years. Their current antipsychotic dosages were converted to chlorpromazine equivalents. All patients were maintained on stable dosages of antipsychotics for at least 3 months before the assessment of TD. Diagnosis of TD was made according to the Research Diagnostic Criteria for TD (RDC-TD) on the basis of AIMS.^21^ To ensure consistency in the ratings of TD, all raters achieved an inter-rater reliability of >0.90 with the investigator bearing overall responsibility for the TD assessments, based on the same series of patients, before commencing the study. We rated the first 7 items of the AIMS to determine the total AIMS score of the abnormal movements. Subjects with two or more two-point ratings, or one or more three-point ratings, on the first seven items of the AIMS were diagnosed as having TD according to RDC-TD.

### Genotyping

Blood samples (5-10 mL) were collected into ethylenediaminetetraacetic acid (EDTA), and genomic DNA was isolated using Accuprep Genomic DNA Extraction Kit (Bioneer, Korea) according to standard procedures. Analysis of genomic DNA was carried out using Polymerase Chain Reaction (PCR) and PCR-based Restriction fragment length polymorphism (PCR-RFLP). PCR amplification was performed with the forward primer 5'-ATC CCC TGC CTC TGT GTG CCA T-3'and the reverse primer 5'-TCA GGA ACT CAG CCC ACA CAG C-3', giving a 404 base pair (bp) product. The PCR product was digested by the restriction enzyme Nia III for approximately 3 hours at 37℃, then the reaction mixture was analyzed by 2% agarose gel electrophoresis to ensure correct amplification of the DNA fragment.^22^ Nia III cleaves the Met81 allele into three fragments of 173, 140, 91 bp, and the Val81 allele into two fragments of 313, 91 bp.

### Statistical analyses

The presence of Hardy-Weinberg equilibrium was tested with the χ^2^ test for goodness of fit. The χ^2^-test was used to compare categorical data and differences for continuous variables were evaluated by using the Student's t-test or analysis of covariance (ANCOVA). The cutoff p value was set at 0.05.

## Results

The genotype frequencies of Val81Met polymorphism of TH gene showed no significant deviation from the Hardy-Weinberg equilibrium (χ^2^=0.46, p=0.50). There were no differences in age (46.82 vs. 44.20, t=1.91, p=0.058), sex distribution (M/F, 48/35 vs. 62/64, χ^2^=1.49, p=0.22), the duration of illness (19.58±8.57 years vs. 19.13±6.28 years, t=0.39, p=0.70), and current antipsychotic dosage in chlorpromazine units (555.44±564.64 vs. 455.83±491.70, t=0.72, p=0.48) between TD and non-TD groups. However, the duration of antipsychotic treatment of the non-TD group was significantly longer than that of the TD group (17.56±5.66 years vs. 15.36±7.56 years, t=2.16, p=0.033). For genetic analysis, there was no significant difference of the TH genotype and allele frequencies between TD and non-TD patients (Table 1). Demographic characteristics and the total AIMS scores according to the TH genotypes were shown in Table 2. Comparison of the TD severity among genotypes of TD patients using ANCOVA with sex, age, duration of antipsychotics and smoking status as covariates revealed no significant difference in the mean total AIMS scores of the different genotypes (F=1.24, p=0.30).

## Discussion

Some studies indicate that conventional neuroleptics may exert a selective neuronal toxicity in the nigrostriatal dopaminergic neurons, and this neurotoxity may underlie extrapyramidal side effects of these drugs.^23^ In agreement with this line of evidence, subchronic haloperidol treatment reduced TH immunostaining in the striatum and caused shrinkage of the dopaminergic cell bodies in substantia nigra.^24^ Lerner et al.^25^ reported changes of TH V_max_ (maximal transport capacity) after chronic haloperidol administration and suggested an elevated TH protein expression by long-term haloperidol treatment. Recently, AMPT, an inhibitor of TH has been reported to be clinically efficacious for treating neuroleptic-induced TD.^12^

Albanese et al.^26^ reported that allelic variations of the tetranucleotide microsatellite (TCAT) in the intron 1 of TH gene alter the functional activity of TH protein. Because the Val81Met polymorphism we have studied was in significant linkage disequilibrium with the TCAT polymorphism, it is also regarded as a functional polymorphism. Therefore, the Val81Met functional polymorphism of TH gene can be regarded as one of the candidate markers for genetic study of individual susceptibility to neuroleptic-induced TD.

Allele frequencies in our sample were similar to those in other Asian samples.^27^ However, in Caucasians the allele frequencies were remarkably different, the Val allele being more frequent.^8^

Polymorphisms generating Val81 and Met81 were compared to the distributions of genotype and allele between the schizophrenic patients with TD and without TD. These comparisons did not support an association between the polymorphism and neuroleptic-induced TD. These results indicate that Val81Met functional polymorphism of TH is not associated with neuroleptic-induced TD in Korean schizophrenic patients.

There are several limitations to generalization of the results of this study. First, although anticholinergics and benzodiazepines have been reported to be beneficial in some cases of TD, in this study we could not control for the prescription of those drugs. Secondly, we cannot exclude presence of population stratification bias. However, we do not think that stratification bias should be considered seriously in our sample because the Korean population is characterized by a genetic homogeneity.^28^ Third, the relatively small sample size limits the generalizationabilty of our findings. Because our sample size is relatively small, our data cannot exclude the possibility that TH Val81Met polymorphism has an influence on susceptibility to TD.

Taking these limitations into account, further investigations involving additional genes and markers and larger samples are warranted to fully understand the genetic pathophysiology of TD.

## Figures and Tables

**TABLE 1:**
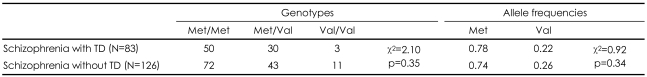
Comparison of the tyrosine hydroxylase Val81Met polymorphism genotypes and allele frequencies between schizophrenia with TD and without TD

TD: tardive dyskinesias

**TABLE 2:**
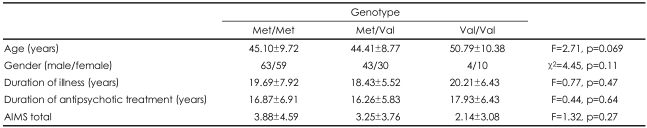
Demographic characteristics and the total AIMS scores according to the TH genotypes

Values are given as mean±SD or n. AIMS: abnormal involuntary movement Scale, TH: tyrosine hydroxylase
